# Supplementary material for: Causal relationships between lipid and glycemic levels in an Indian population: A bidirectional Mendelian randomization approach
Source: PLoS One. 2020 Jan 29;15(1):e0228269. doi: 10.1371/journal.pone.0228269 (PMC6988960; doi:10.1371/journal.pone.0228269)
Supplement: S1 Appendix — (DOCX) [file pone.0228269.s001.docx]

**Supplementary Information**

**Study Design**: The Indian Migration Study (IMS) was established in 2005-2007 to investigate the effects of rural-to-urban migration on cardiometabolic risk in India. It used a sib-pair design, comparing chronic disease risk factors in migrant urban factory workers and their spouses with that of their siblings who still lived in rural areas (non-migrants). Factory workers and their spouse residing in four cities were recruited and asked to invite their related sibling, of preferably same sex and similar age, still residing in the place of origin, thereby covering over 20 states of India.

**Phenotypes**: Information on age, gender, site (city) and location (urban/rural) was collected at the time of interview. Standing height was measured using a plastic stadiometer (Leicester height measure; Chasmors Ltd) and weight was measured in light clothes with shoes off using a digital scale with an accuracy of 100 g (Model PS16, Beurer, Germany) [1]. The measurements were made twice and an average was taken. Difference of ≤ 0.5 cm for height and ≤ 0.5 kg for weight between the two readings was considered acceptable. Body Mass Index (BMI) was calculated using the above information. Diet was assessed by an interviewer-administered food frequency questionnaire (FFQ) collecting details on consumption of 184 different food items (daily, weekly, monthly or yearly). Average daily fat intake and average daily carbohydrate intake were calculated in g/day, average daily intake of energy consumption as kcal/day. Physical activity of the past month was assessed using an interviewer-administered questionnaire collecting data on several activities and calculated as MET hours per day. Average time spent and frequency of activity was documented for each activity. “Metabolic equivalent tasks (METs) were estimated as the ratio of resting metabolic rate where 1 MET is equivalent to the energy expenditure value of sitting quietly”. Alcohol consumption and smoking were recorded as categorical responses using the questionnaire.

**Biochemical Parameters**: Fasting blood samples were collected from the participants, stored at -20°C locally and transported monthly to AIIMS, New Delhi for analyses. Serum samples were used for generating data on lipid profile – HDL-C, total cholesterol and triglycerides. Estimation of HDL-C was done using elimination method; total cholesterol was estimated using enzymatic endpoint method and triglycerides by GPO-PAP method using kits available from Randox Laboratory Ltd. (Crumlin City, United Kingdom). The quality control of local assays was performed with regular external standards and internal duplicate assays and monitored by AIIMS. For quality assurance, the Cardiac Biochemistry Lab, AIIMS is part of the UK National External Quality Assessment (<http://www.ukneqas.org.uk/>).

Fasting plasma glucose was estimated on the day of blood collection in the local lab at the site using the GOD-PAP method and RANDOX kits (Randox Laboratories, Crumlin, UK) [2]. Fasting plasma insulin level was estimated using kits from MERCODIA (Mercodia AB, Sylveniusgatan, Uppsala, Sweden) by ELISA method [3]. The HOMA scores were further validated in Indian setting by comparing with biochemical markers in healthy Indian people. Diabetes was identified as fasting blood glucose ≥7mmol/l or if detected earlier (with or without medication).

**Genotyping**: Sequenom Mass ARRAY technology was used for genotyping the SNPs related to glycemic traits and lipid traits along with the two multiple pools of a total of 59 SNPs in Centre for Cellular and Molecular Biology (CCMB), Hyderabad. The considered SNPs were associated with the traits at the genome wide levels of significance in European studies and reported upto the year 2008. The SNPs were also considered for biological plausibility. Some SNPs with p values >1×10−7 were also included in the sample to validate their associations in the Indian setting. The resultant genotyping success rate was >95% and duplicate samples (10% duplicates in each 96-well plate) resulted in >97% concordance.

**Statistical Analyses:** After adjusting for clustering (family/sibling effect), continuous variables were summarized as mean and standard deviation (SD) and categorical variables as number of participants and percentages. We used t-test and χ^2^ test to compare the distribution of covariates according to sex, location (urban/rural) and site (city).

For deriving genetic risk scores to be used as instrumental variables in IV analyses, the significant SNPs for lipid and glycemic levels were examined for association with forced confounders (age, sex, site, location and BMI) in Model-1 (Table H). The SNPs were also examined for association with the additional lifestyle confounders (diet, physical activity, alcohol use and smoking) that were accounted in Model 2. Similarly, to rule out pleiotropic effect of SNPs, the significant SNPs related to the exposures were tested for association with outcomes on the causal pathways to make sure that SNPs (to be used as proxies for exposures) are not associated with outcomes by any other route other than that of exposures itself. Therefore, SNPs of exposures were tested for association with outcomes using mixed linear model in the first step and then adjusted for the exposure of interest in the second step (Tables F and G). For example, association between lipid related SNP (instrument) and glycemic trait (outcome) was tested in the first step and then adjusted for the respective lipid trait (exposure) in the second step. Only if the significant SNPs were not associated with any outcome (after adjusting for exposure) and/or confounders, it was used for instrument generation and for instrument variable (IV) analyses for BMR. The validated SNPs that met all the MR assumptions were combined to derive the weighted genetic risk scores (wGRS) which were used as instruments in IV analyses. In order to meet the assumptions of MR analysis, association of the instrument with the respective exposure, confounder and outcome adjusted for the exposure was checked, before performing the BMR. 2 Stage Least Square (2SLS) method was used for performing IV analyses wherein, in the first stage, the standardized parameters were regressed on the respective SNP/risk scores (e.g. standardized glycemic traits on instruments identified for each trait and similarly for lipid traits). Thereafter, in the second stage, the response of interest (e.g. the standardized glycemic and the lipid parameters) was regressed on the fitted values of the predictor obtained from the first stage. The interpretation was made as SD change in the outcome with per risk allele/score increase.

**Association between Lipids and Glycemic Levels:** Total cholesterol was observed to be associated with HOMA-IR (β=0.03, SE=0.01, p=0.031) and fasting glucose (β=0.03, SE=0.01, p=0.009) (Table B). Triglyceride level was associated with all the glycemic traits [HOMA-IR (β=0.11, SE=0.01, p<0.001), HOMA-β (β=0.08, SE=0.01, p<0.001), fasting insulin (β=0.11, SE=0.01, p<0.001) and fasting glucose (β=0.05, SE=0.01, p<0.001)] (Table B). HDL-C was associated only with fasting glucose (β=0.03, SE=0.01, p=0.012) (Table B). However, LDL-C was not seen to be associated with any of the glycemic parameters. In the reverse direction, HOMA-IR was found to be associated with Total Cholesterol (β=0.03, SE=0.03, p=0.032) and Triglycerides (β=0.12, SE=0.01, p<0.001) (Table A). Triglycerides, alone, showed significant association with HOMA-β (β=0.08, SE=0.01, p<0.001) and Fasting Insulin (β=0.11, SE=0.01, p<0.001) (Table A). Fasting glucose was observed to be associated with all the lipid traits, except LDL-C [total cholesterol (β=0.04, SE=0.02, p=0.007), triglycerides (β=0.05, SE=0.01, p<0.001 and HDL-C (β=0.04, SE=0.01, p=0.004)] (Table A).

**Genetic Variants of Glycemic and Lipid levels:** Out of 44 SNPs, 5 SNPs deviated from HWE - rs864745 at *JAZF1* locus, rs5015480 at *HHEX* locus, rs7923837 at *HHEX* locus, rs9402571 at *SGK1* locus and rs12255372 at *TCF7L2* locus (Table C). Out of the 9 lipid SNPs, 3 SNPs (rs662799 at *APOAV*, rs562338 at *APOB* and rs6511720 at *LDLR*) were validated for total cholesterol (Table D). However, rs662799 was found to be significantly associated with HOMA-β conditional on total cholesterol (Table F). Thus, 2 separate wGRS were generated for total cholesterol, one using all the 3 SNPs (not to be used for analyzing causal association between HOMA-β and total cholesterol) and the second after excluding rs662799. Further, rs6511720and rs562338werefound to be associated with physical activity and daily fat intake, respectively (Table H) that were treated as confounders in Model-2 (Table 2).5 SNPs were found to be associated with Triglycerides (rs662799 at *APOAV*, rs780094 at *GCKR*, rs4420638 at *APOE/C1/C4*, rs6993414 and rs10503669 at *LPL*) (Table D). The two LPL variants were found to be associated with location (forced confounder of Model-1) (Table H), hence they were not included in the risk score generation. Therefore, wGRS for triglycerides was generated using the other 3 SNPs. Single SNP, rs6993414 at *LPL* which was found to be associated with HDL-C (Table D) was also found to be associated with HOMA-IR, HOMA-β and Fasting Insulin, conditional on exposure (Table F) and also with a forced confounder (location) (Table H). Hence HDL-C instrument was completely excluded from the causal analysis. wGRS for LDL-C was generated using 2 validated SNPs rs562338 at *APOB* and rs6511720 at *LDLR* (Table D). However, similar to total cholesterol, these variants were also found to be associated with physical activity and daily fat intake.

For glycemic traits, 3 SNPs (rs2641348 at *ADAM30*, rs10811661 at *CDKN2A/* and rs10923931 at *NOTCH2*) were found to be associated with HOMA-IR and fasting insulin (Table E). Except rs10923931, which was found to be associated with a forced confounder (site) (Table H), the other two SNPs were used to generate wGRS for HOMA-IR and fasting insulin. Only one SNP, rs2641348 at *ADAM30* was found to be associated with HOMA-β (Table E), which was also used as an instrument for HOMA-β. Two SNPs**,** rs7756992 at *CDKAL1* and rs7903146 at *TCF7L2*, were associated with fasting glucose (Table E), which were used for generating wGRS for fasting glucose. None of the glycemic SNPs was found to be associated with lipid traits (outcomes) except through the route of exposures (glycemic traits) (Table G). However, rs7756992 was found to be associated with smoking (Table H) that was used in the instrument for fasting glucose.

**References**

## 1. Ebrahim S, Kinra S, Bowen L, Andersen E, Ben-Shlomo Y, Lyngdoh T, Ramakrishnan L, Ahuja RC, Joshi P, Das SM, Mohan M, Davey Smith G, Prabhakaran D, Reddy KS, Indian Migration Study g: The effect of rural-to-urban migration on obesity and diabetes in India: a cross-sectional study. PLoS medicine 2010;7:e1000268

## 2. Trinder P: Determination of blood glucose using 4-amino phenazone as oxygen acceptor. Journal of clinical pathology 1969;22:246

## 3. Sjostrand M, Gudbjornsdottir S, Strindberg L, Lonnroth P: Delayed transcapillary delivery of insulin to muscle interstitial fluid after oral glucose load in obese subjects. Diabetes 2005;54:2266

## Supplementary Tables

**Table A: Association of Glycemic traits (independent variable) with lipid traits (dependent variable)**

|  |  | **HOMA-IR** | | **HOMA-β** | | **Fasting Insulin** | | **Fasting Glucose** | |
| --- | --- | --- | --- | --- | --- | --- | --- | --- | --- |
|  |  | **β (SE)** | **p-value** | **β (SE)** | **p-value** | **β (SE)** | **p-value** | **β (SE)** | **p-value** |
| **Total Cholesterol** | Model^1^ | 0.03 (0.02) | 0.032^*^ | 0.01 (0.05) | 0.455 | 0.03 (0.02) | 0.067 | 0.04 (0.02) | 0.007^*^ |
|  | Model^2^ | 0.03 (0.02) | 0.021^*^ | 0.01 (0.02) | 0.327 | 0.03 (0.02) | 0.043 | 0.04 (0.02) | 0.013^*^ |
| **Triglycerides** | Model^1^ | 0.12 (0.01) | <0.001^*^ | 0.08 (0.01) | <0.001^*^ | 0.11 (0.01) | <0.001^*^ | 0.05 (0.01) | <0.001^*^ |
|  | Model^2^ | 0.12 (0.01) | <0.001^*^ | 0.08 (0.01) | <0.001^*^ | 0.11 (0.01) | <0.001^*^ | 0.05 (0.01) | 0.001^*^ |
| **HDL-C** | Model^1^ | 0.03 (0.01) | 0.075 | -0.01 (0.01) | 0.620 | 0.02 (0.01) | 0.166 | 0.04 (0.01) | 0.004^*^ |
|  | Model^2^ | 0.03 (0.01) | 0.039^*^ | 0.00 (0.01) | 0.956 | 0.03 (0.01) | 0.087 | 0.04 (0.02) | 0.008^*^ |
| **LDL-C** | Model^1^ | 0.00 (0.01) | 0.813 | -0.01 (0.01) | 0.634 | -0.01 (0.01) | 0.692 | 0.02 (0.02) | 0.231 |
|  | Model^2^ | 0.00 (0.01) | 0.910 | -0.01 (0.01) | 0.721 | 0.00 (0.01) | 0.797 | 0.02 (0.02) | 0.266^[[1]](#footnote-1)^ |

**Table B: Association of Lipid traits (independent variable) with Glycemic traits (dependent variable)**

|  |  | **Total Cholesterol** | | **Triglycerides** | | **HDL-C** | | **LDL-C** | |
| --- | --- | --- | --- | --- | --- | --- | --- | --- | --- |
|  |  | **β (SE)** | **p-value** | **β (SE)** | **p-value** | **β (SE)** | **p-value** | **β (SE)** | **p-value** |
| **HOMA-IR** | Model^1^ | 0.03 (0.01) | 0.031^*^ | 0.11 (0.01) | <0.001^*^ | 0.03 (0.01) | 0.075 | 0.00 (0.01) | 0.820 |
|  | Model^2^ | 0.03 (0.01) | 0.021^*^ | 0.11 (0.01) | <0.001^*^ | 0.03 (0.01) | 0.037^*^ | -0.00 (0.01) | 0.912 |
| **HOMA-B** | Model^1^ | 0.01 (0.01) | 0.384 | 0.08 (0.01) | <0.001^*^ | -0.01 (0.02) | 0.604 | -0.01 (0.01) | 0.695 |
|  | Model^2^ | 0.02 (0.01) | 0.279 | 0.08 (0.01) | <0.001^*^ | -0.01 (0.01) | 0.932 | -0.01 (0.01) | 0.771 |
| **Fasting Insulin** | Model^1^ | 0.03 (0.01) | 0.065 | 0.11 (0.01) | <0.001^*^ | 0.02 (0.01) | 0.167 | -0.01 (0.01) | 0.701 |
|  | Model^2^ | 0.03 (0.02) | 0.043^*^ | 0.11 (0.01) | <0.001^*^ | 0.02 (0.01) | 0.084 | -0.01 (0.01) | 0.800 |
| **Fasting Glucose** | Model^1^ | 0.03 (0.01) | 0.009^*^ | 0.05 (0.01) | <0.001^*^ | 0.03 (0.01) | 0.012^*^ | 0.01 (0.01) | 0.284 |
|  | Model^2^ | 0.03 (0.01) | 0.017^*^ | 0.05 (0.01) | <0.001^*^ | 0.03 (0.01) | 0.020^*^ | 0.01 (0.01) | 0.337^[[2]](#footnote-2)^ |

**Table C: Minor Allele Frequency and Hardy-Weinberg Equilibrium test of studied SNPs in IMS sample**

| SNP | | Loci | N^#^ | | MAF  (Risk Allele) | Bangalore | Hyderabad | Nagpur | Lucknow | Overall |
| --- | --- | --- | --- | --- | --- | --- | --- | --- | --- | --- |
| SNPs related to Lipids | | | | | | | | | | |
| rs562338 | | ***APOB*** | 2417 | | 0.1318 (T) | 0.678 | 0.035 | 0.194 | 0.278 | 0.010 |
| rs662799 | | ***APOAV*** | 2411 | | 0.1908 (G) | 0.030 | 0.526 | 1.000 | 0.174 | 0.598 |
| rs780094 | | ***GCKR*** | 2435 | | 0.2136 (A) | 0.204 | 0.635 | 0.341 | 0.080 | 0.025 |
| rs1864163 | | ***CETP*** | 2400 | | 0.2002 (A) | 0.058 | 0.456 | 0.456 | 0.905 | 0.445 |
| rs4420638 | | ***APOE/C1/C4*** | 2424 | | 0.1273 (G) | 0.152 | 0.030 | 0.587 | 1.000 | 0.927 |
| rs4775041 | | ***LIPC*** | 2433 | | 0.2067 (C) | 0.071 | 0.898 | 0.908 | 0.505 | 0.266 |
| rs6511720 | | ***LDLR*** | 2407 | | 0.0638 (T) | 1.000 | 0.719 | 0.089 | 0.555 | 0.610 |
| rs6993414 | | ***LPL*** | 2416 | | 0.1258 (G) | 0.281 | 0.127 | 0.868 | 0.841 | 0.228 |
| rs10503669 | | ***LPL*** | 2442 | | 0.1122 (A) | 0.104 | 0.273 | 0.560 | 0.077 | 0.011 |
| SNPs related to T2D | | | | | | | | | | |
| rs757210 | ***TCF2*** | | | 2414 | 0.2865 (A) | 0.098 | 0.703 | 0.097 | 0.481 | 0.059 |
| rs864745 | ***JAZF1*** | | | 1948 | 0.2710 (G) | 0.397 | **<0.001**^*^ | **<0.001**^*^ | 0.832 | **<0.001**^*^ |
| rs932206 | ***CXCR4*** | | | 2432 | 0.1423 (A) | 0.499 | 0.077 | 0.454 | 1.000 | 0.081 |
| rs1055080 | ***Foxa2*** | | | 2421 | 0.1012 (A) | 0.810 | 0.027 | 0.851 | 0.641 | 0.436 |
| rs1111875 | ***HHEX*** | | | 2415 | 0.4124 (G) | 0.756 | 0.614 | 0.275 | 0.643 | 0.314 |
| rs1153188 | ***DCD*** | | | 2414 | 0.1972(A) | 0.465 | 0.009 | 0.642 | 0.482 | 0.404 |
| rs1256517 | ***LOC646279*** | | | 2420 | 0.1262 (C) | 0.020 | 0.479 | 0.728 | 0.606 | 0.645 |
| rs1799854 | ***ABCC8*** | | | 2432 | 0.3250 (T) | 0.197 | 0.629 | 1.000 | 0.677 | 0.230 |
| rs1801282 | ***PPARG2*** | | | 2438 | 0.1300 (G) | 1.000 | 0.009 | 1.000 | 0.869 | 0.152 |
| rs2237892 | ***KCNQ1*** | | | 2434 | 0.0101 (T) | 1.000 | 1.000 | 0.076 | 0.083 | 0.023 |
| rs2268573 | ***GCK*** | | | 2419 | 0.4450 (C) | 0.843 | 0.008 | 1.000 | 0.939 | 0.128 |
| rs2476601 | ***PTPN22*** | | | 2444 | 0.008 (A) | 1.000 | 1.000 | 1.000 | 1.000 | 1.000 |
| rs2641348 | ***ADAM30*** | | | 2400 | 0.2375 (C) | 0.343 | 0.072 | 0.296 | 0.911 | 1.000 |
| rs2876711 | ***KCTD12*** | | | 2402 | 0.3004 (C) | 0.090 | 0.073 | 0.474 | 0.649 | 0.734 |
| rs3856806 | ***PPARG2*** | | | 2436 | 0.1461 (T) | 0.726 | 0.183 | 0.592 | 0.877 | 0.417 |
| rs5015480 | ***HHEX*** | | | 2309 | 0.4504 (C) | 0.307 | 0.870 | 0.001^*^ | 0.330 | 0.048 |
| rs6698181 | ***PKN2*** | | | 2416 | 0.1743 (T) | 0.059 | 0.007 | 0.003 | 0.517 | 0.011 |
| rs7578597 | ***THADA*** | | | 2425 | 0.1324 (C) | 0.283 | 1.000 | 0.014 | 0.733 | 0.051 |
| rs7754840 | ***CDKAL1*** | | | 2442 | 0.2643 (C) | 0.424 | 0.758 | 0.090 | 0.628 | 0.876 |
| rs7756992 | ***CDKAL1*** | | | 2420 | 0.2626 (G) | 0.225 | 0.481 | 0.557 | 0.437 | 0.529 |
| rs7903146 | ***TCF7L2*** | | | 2427 | 0.2843 (T) | 0.171 | 1.000 | 0.022 | 0.109 | 0.008 |
| rs7923837 | ***HHEX*** | | | 2404 | 0.4495 (G) | 0.619 | 0.000^*^ | 0.063 | 0.125 | 1.000 |
| rs7961581 | ***TSPAN8,LGR5*** | | | 2360 | 0.3320 (C) | 0.738 | 0.932 | 0.017 | 0.789 | 0.086 |
| rs9402571 | ***SGK1*** | | | 2329 | 0.2258 (G) | 1.000 | 0.205 | 0.003 | 0.001^*^ | 0.000^*^ |
| rs9472138 | ***VEGFA*** | | | 2423 | 0.1562 (T) | 0.859 | 0.430 | 0.882 | 0.889 | 0.538 |
| rs10010131 | ***WFS1*** | | | 2438 | 0.2799 (A) | 0.810 | 0.503 | 0.083 | 0.108 | 0.024 |
| rs10490072 | ***BCL11A*** | | | 2439 | 0.0945 (C) | 0.746 | 1.000 | 1.000 | 0.030 | 0.154 |
| rs10811661 | ***CDKN2A/B*** | | | 2420 | 0.1502 (C) | 0.348 | 0.546 | 0.003 | 0.305 | 0.056 |
| rs10823406 | ***NGN3*** | | | 2439 | 0.2247 (A) | 0.578 | 0.643 | 0.310 | 0.603 | 0.684 |
| rs10923931 | ***NOTCH2*** | | | 2419 | 0.2191 (T) | 0.197 | 0.440 | 0.104 | 0.463 | 0.137 |
| rs10946398 | ***CDKAL1*** | | | 2427 | 0.2490 (C) | 0.138 | 1.000 | 0.180 | 0.771 | 0.958 |
| rs12255372 | ***TCF7L2*** | | | 2434 | 0.2132 (T) | 0.048 | 0.429 | 0.002 | 0.607 | 0.001^*^ |
| rs12779790 | ***CDC123,CAMK1D*** | | | 2420 | 0.1295 (G) | 0.847 | 0.713 | 0.081 | 0.608 | 0.242 |
| rs13266634 | ***SLC30A8*** | | | 2436 | 0.2118 (T) | 0.687 | 0.360 | 1.000 | 0.443 | 0.856 |
| rs17044137 | ***FLJ39370*** | | | 2420 | 0.1091 (A) | 0.776 | 0.045 | 0.058 | 0.307 | 0.005 |
|  |  | | |  |  |  |  |  |  | ^[[3]](#footnote-3)^ |

**Table D: Validation of lipid related SNPs in IMS**

|  |  |  | **Total Cholesterol** | | **Triglycerides** | | **HDL-C** | | **LDL-C** | |
| --- | --- | --- | --- | --- | --- | --- | --- | --- | --- | --- |
| **SNP** | ***Loci*** |  | **β (SE)** | **p** | **β (SE)** | **P** | **β (SE)** | **P** | **β (SE)** | **P** |
| **rs562338** | ***APOB*** | Model^1^ | -0.11 (0.05) | 0.016**^*^** | -0.06 (0.05) | 0.199 | 0.05 (0.05) | 0.305 | -0.11 (0.05) | 0.015**^*^** |
|  |  | Model^2^ | -0.11 (0.05) | 0.015**^*^** | -0.06 (0.05) | 0.233 | 0.05 (0.05) | 0.292 | -0.12 (0.05) | 0.012**^*^** |
| **rs662799** | ***APOAV*** | Model^1^ | 0.09 (0.04) | 0.023**^*^** | 0.17 (0.04) | <0.001**^*^** | -0.04 (0.04) | 0.327 | 0.07 (0.04) | 0.095 |
|  |  | Model^2^ | 0.10 (0.04) | 0.013**^*^** | 0.17 (0.04) | <0.001**^*^** | -0.03 (0.04) | 0.455 | 0.08 (0.04) | 0.066 |
| **rs780094** | ***GCKR*** | Model^1^ | 0.05 (0.04) | 0.153 | 0.08 (0.04) | 0.048**^*^** | -0.04 (0.04) | 0.352 | 0.06 (0.04) | 0.135 |
|  |  | Model^2^ | 0.04 (0.04) | 0.242 | 0.07 (0.04) | 0.073 | -0.04 (0.04) | 0.300 | 0.05 (0.04) | 0.198 |
| **rs1864163** | ***CETP*** | Model^1^ | 0.01 (0.04) | 0.883 | 0.00 (0.04) | 0.969 | 0.04 (0.04) | 0.329 | 0.00 (0.04) | 0.984 |
|  |  | Model^2^ | 0.00 (0.04) | 0.913 | 0.00 (0.04) | 0.982 | 0.04 (0.04) | 0.354 | 0.00 (0.04) | 0.995 |
| **rs4420638** | ***APOE/C1/C4*** | Model^1^ | 0.04 (0.05) | 0.372 | 0.10 (0.05) | 0.048**^*^** | -0.09 (0.05) | 0.067 | 0.03 (0.05) | 0.540 |
|  |  | Model^2^ | 0.05 (0.05) | 0.291 | 0.10 (0.05) | 0.042**^*^** | -0.08 (0.05) | 0.076 | 0.04 (0.05) | 0.429 |
| **rs4775041** | ***LIPC*** | Model^1^ | 0.05 (0.04) | 0.216 | 0.03 (0.04) | 0.411 | 0.07 (0.04) | 0.064 | 0.03 (0.04) | 0.456 |
|  |  | Model^2^ | 0.05 (0.04) | 0.226 | 0.02 (0.04) | 0.539 | 0.07 (0.04) | 0.060 | 0.03 (0.04) | 0.451 |
| **rs6511720** | ***LDLR*** | Model^1^ | -0.14 (0.06) | 0.026**^*^** | -0.06 (0.06) | 0.369 | 0.09 (0.06) | 0.142 | -0.13 (0.06) | 0.033**^*^** |
|  |  | Model^2^ | -0.13 (0.06) | 0.042**^*^** | -0.05 (0.06) | 0.432 | 0.09 (0.06) | 0.139 | -0.12 (0.06) | 0.050 |
| **rs6993414** | ***LPL*** | Model^1^ | -0.02 (0.05) | 0.612 | -0.12 (0.05) | 0.013**^*^** | 0.10 (0.05) | 0.038**^*^** | -0.02 (0.05) | 0.636 |
|  |  | Model^2^ | -0.03 (0.05) | 0.508 | -0.13 (0.05) | 0.010**^*^** | 0.09 (0.05) | 0.055 | -0.03 (0.05) | 0.555 |
| **rs10503669** | ***LPL*** | Model^1^ | -0.01 (0.05) | 0.889 | -0.12 (0.05) | 0.018**^*^** | 0.10 (0.05) | 0.060 | -0.01 (0.05) | 0.926 |
|  |  | Model^2^ | 0.00 (0.05) | 0.980 | -0.12 (0.05) | 0.019**^*^** | 0.09 (0.05) | 0.067 | 0.00 (0.05) | 0.982^[[4]](#footnote-4)^ |

**Table E: Validation of glycemic related SNPs in IMS**

|  |  |  | **HOMA-IR** | | **HOMA-β** | | **Fasting Insulin** | | **Fasting Glucose** | |
| --- | --- | --- | --- | --- | --- | --- | --- | --- | --- | --- |
| **SNP** | **Loci** |  | **β (SE)** | **p** | **β (SE)** | **P** | **β (SE)** | **p** | **β (SE)** | **P** |
| **rs757210** | ***TCF2*** | Model^1^ | 0.06 (0.03) | 0.088 | 0.06 (0.03) | 0.061 | 0.06 (0.03) | 0.071 | -0.01 (0.03) | 0.668 |
|  |  | Model^2^ | 0.05 (0.03) | 0.123 | 0.06 (0.03) | 0.089 | 0.05 (0.03) | 0.102 | -0.01 (0.03) | 0.690 |
| **rs932206** | ***CXCR4*** | Model^1^ | -0.02 (0.04) | 0.579 | 0.02 (0.04) | 0.554 | -0.01 (0.04) | 0.759 | -0.07 (0.04) | 0.076 |
|  |  | Model^2^ | -0.03 (0.04) | 0.493 | 0.02 (0.04) | 0.659 | -0.02 (0.04) | 0.660 | -0.07 (0.04) | 0.070 |
| **rs1055080** | ***Foxa2*** | Model^1^ | 0.02 (0.05) | 0.664 | 0.02 (0.05) | 0.633 | 0.03 (0.05) | 0.609 | -0.03 (0.05) | 0.562 |
|  |  | Model^2^ | 0.03 (0.05) | 0.512 | 0.03 (0.05) | 0.561 | 0.04 (0.05) | 0.478 | -0.02 (0.05) | 0.736 |
| **rs1111875** | ***HHEX*** | Model^1^ | -0.02 (0.03) | 0.548 | -0.04 (0.03) | 0.227 | -0.02 (0.03) | 0.433 | 0.04 (0.03) | 0.186 |
|  |  | Model^2^ | -0.02 (0.03) | 0.541 | -0.03 (0.03) | 0.266 | -0.02 (0.03) | 0.437 | 0.03 (0.03) | 0.240 |
| **rs1153188** | ***DCD*** | Model^1^ | -0.04 (0.04) | 0.340 | -0.01 (0.04) | 0.835 | -0.03 (0.04) | 0.372 | -0.02 (0.03) | 0.579 |
|  |  | Model^2^ | -0.04 (0.04) | 0.311 | -0.01 (0.04) | 0.894 | -0.04 (0.04) | 0.356 | -0.03 (0.03) | 0.445 |
| **rs1256517** | ***LOC646279*** | Model^1^ | -0.03 (0.05) | 0.558 | -0.04 (0.04) | 0.312 | -0.04 (0.05) | 0.423 | 0.07 (0.04) | 0.091 |
|  |  | Model^2^ | -0.03 (0.05) | 0.482 | -0.05 (0.04) | 0.234 | -0.04 (0.05) | 0.351 | 0.07 (0.04) | 0.078 |
| **rs1799854** | ***ABCC8*** | Model^1^ | 0.00 (0.03) | 0.915 | 0.00 (0.03) | 0.878 | 0.00 (0.03) | 0.944 | -0.01 (0.03) | 0.852 |
|  |  | Model^2^ | 0.00 (0.03) | 0.909 | 0.01 (0.03) | 0.770 | 0.01 (0.03) | 0.889 | 0.00 (0.03) | 0.957 |
| **rs1801282** | ***PPARG2*** | Model^1^ | -0.01 (0.05) | 0.777 | 0.02 (0.05) | 0.724 | -0.01 (0.05) | 0.889 | -0.05 (0.04) | 0.258 |
|  |  | Model^2^ | -0.03 (0.05) | 0.590 | 0.01 (0.05) | 0.925 | -0.02 (0.05) | 0.683 | -0.05 (0.04) | 0.270 |
| **rs2237892** | ***KCNQ1*** | Model^1^ | -0.03 (0.13) | 0.793 | -0.10 (0.13) | 0.439 | -0.04 (0.13) | 0.775 | 0.02 (0.12) | 0.859 |
|  |  | Model^2^ | -0.03 (0.13) | 0.839 | -0.09 (0.13) | 0.504 | -0.03 (0.13) | 0.832 | 0.01 (0.12) | 0.924 |
| **rs2268573** | ***GCK*** | Model^1^ | 0.03 (0.03) | 0.341 | 0.02 (0.03) | 0.459 | 0.03 (0.03) | 0.402 | 0.03 (0.03) | 0.238 |
|  |  | Model^2^ | 0.03 (0.03) | 0.313 | 0.03 (0.03) | 0.297 | 0.03 (0.03) | 0.344 | 0.02 (0.03) | 0.392 |
| **rs2641348** | ***ADAM30*** | Model^1^ | -0.09 (0.04) | 0.018**^*^** | -0.07 (0.04) | 0.049**^*^** | -0.09 (0.04) | 0.013**^*^** | 0.01 (0.03) | 0.665 |
|  |  | Model^2^ | -0.09 (0.04) | 0.017^*^ | -0.07 (0.04) | 0.059 | -0.09 (0.04) | 0.013**^*^** | 0.01 (0.03) | 0.682 |
| **rs2876711** | ***KCTD12*** | Model^1^ | -0.02 (0.03) | 0.525 | -0.04 (0.03) | 0.265 | -0.02 (0.03) | 0.488 | 0.01 (0.03) | 0.833 |
|  |  | Model^2^ | -0.02 (0.03) | 0.514 | -0.03 (0.03) | 0.394 | -0.02 (0.03) | 0.517 | -0.01 (0.03) | 0.823 |
| **rs3856806** | ***PPARG2*** | Model^1^ | 0.01 (0.04) | 0.745 | 0.04 (0.04) | 0.326 | 0.02 (0.04) | 0.644 | -0.04 (0.04) | 0.323 |
|  |  | Model^2^ | 0.01 (0.04) | 0.785 | 0.03 (0.04) | 0.440 | 0.02 (0.04) | 0.713 | -0.03 (0.04) | 0.484 |
| **rs6698181** | ***PKN2*** | Model^1^ | -0.03 (0.04) | 0.465 | 0.01 (0.04) | 0.834 | -0.02 (0.04) | 0.553 | -0.04 (0.04) | 0.338 |
|  |  | Model^2^ | -0.03 (0.04) | 0.459 | 0.01 (0.04) | 0.742 | -0.02 (0.04) | 0.559 | -0.04 (0.04) | 0.271 |
| **rs7578597** | ***THADA*** | Model^1^ | 0.00 (0.04) | 0.945 | 0.01 (0.04) | 0.901 | 0.00 (0.04) | 0.924 | 0.01 (0.04) | 0.738 |
|  |  | Model^2^ | 0.00 (0.04) | 0.917 | 0.01 (0.04) | 0.842 | 0.00 (0.04) | 0.952 | 0.02 (0.04) | 0.613 |
| **rs7754840** | ***CDKAL1*** | Model^1^ | 0.03 (0.03) | 0.421 | -0.01 (0.03) | 0.664 | 0.02 (0.03) | 0.509 | 0.04 (0.03) | 0.200 |
|  |  | Model^2^ | 0.03 (0.03) | 0.337 | -0.01 (0.03) | 0.774 | 0.03 (0.04) | 0.409 | 0.04 (0.03) | 0.224 |
| **rs7756992** | ***CDKAL1*** | Model^1^ | 0.01 (0.03) | 0.786 | -0.06 (0.03) | 0.068 | 0.00 (0.03) | 0.945 | 0.08 (0.03) | 0.008**^*^** |
|  |  | Model^2^ | 0.02 (0.04) | 0.655 | -0.06 (0.03) | 0.101 | 0.00 (0.04) | 0.903 | 0.08 (0.03) | 0.011**^*^** |
| **rs7903146** | ***TCF7L2*** | Model^1^ | 0.02 (0.03) | 0.600 | -0.01 (0.03) | 0.645 | 0.01 (0.03) | 0.781 | 0.06 (0.03) | 0.049**^*^** |
|  |  | Model^2^ | 0.02 (0.03) | 0.663 | -0.02 (0.03) | 0.559 | 0.01 (0.03) | 0.861 | 0.06 (0.03) | 0.042**^*^** |
| **rs7961581** | ***TSPAN8,LGR5*** | Model^1^ | -0.03 (0.03) | 0.334 | -0.03 (0.03) | 0.394 | -0.03 (0.03) | 0.320 | 0.00 (0.03) | 0.985 |
|  |  | Model^2^ | -0.04 (0.03) | 0.278 | -0.03 (0.03) | 0.325 | -0.04 (0.03) | 0.261 | 0.00 (0.03) | 0.943 |
| **rs9472138** | ***VEGFA*** | Model^1^ | -0.06 (0.04) | 0.124 | -0.06 (0.04) | 0.125 | -0.06 (0.04) | 0.127 | -0.01 (0.04) | 0.743 |
|  |  | Model^2^ | -0.07 (0.04) | 0.109 | -0.06 (0.04) | 0.114 | -0.07 (0.04) | 0.112 | -0.01 (0.04) | 0.753 |
| **rs10010131** | ***WFS1*** | Model^1^ | 0.01 (0.04) | 0.837 | 0.00 (0.03) | 0.951 | 0.01 (0.04) | 0.834 | 0.00 (0.03) | 0.901 |
|  |  | Model^2^ | 0.01 (0.04) | 0.777 | 0.01 (0.03) | 0.791 | 0.01 (0.04) | 0.760 | -0.01 (0.03) | 0.843 |
| **rs10490072** | ***BCL11A*** | Model^1^ | -0.05 (0.05) | 0.346 | -0.04 (0.05) | 0.377 | -0.05 (0.05) | 0.314 | 0.02 (0.05) | 0.677 |
|  |  | Model^2^ | -0.06 (0.05) | 0.260 | -0.05 (0.05) | 0.313 | -0.06 (0.05) | 0.239 | 0.01 (0.05) | 0.802 |
| **rs10811661** | ***CDKN2A/B*** | Model^1^ | -0.09 (0.04) | 0.037**^*^** | -0.08 (0.04) | 0.065 | -0.09 (0.04) | 0.036**^*^** | -0.02 (0.04) | 0.688 |
|  |  | Model^2^ | -0.10 (0.04) | 0.025**^*^** | -0.08 (0.04) | 0.044**^*^** | -0.10 (0.04) | 0.023**^*^** | -0.01 (0.04) | 0.710 |
| **rs10823406** | ***NGN3*** | Model^1^ | -0.02 (0.04) | 0.638 | -0.02 (0.03) | 0.619 | -0.02 (0.04) | 0.628 | 0.00 (0.03) | 0.958 |
|  |  | Model^2^ | -0.02 (0.04) | 0.626 | -0.02 (0.04) | 0.562 | -0.02 (0.04) | 0.606 | 0.01 (0.03) | 0.879 |
| **rs10923931** | ***NOTCH2*** | Model^1^ | -0.07 (0.04) | 0.045**^*^** | -0.05 (0.04) | 0.187 | -0.08 (0.04) | 0.039**^*^** | 0.00 (0.03) | 0.974 |
|  |  | Model^2^ | -0.07 (0.04) | 0.050 | -0.05 (0.04) | 0.194 | -0.08 (0.04) | 0.042**^*^** | 0.01 (0.03) | 0.876 |
| **rs10946398** | ***CDKAL1*** | Model^1^ | 0.03 (0.04) | 0.329 | -0.01 (0.03) | 0.748 | 0.03 (0.04) | 0.404 | 0.04 (0.03) | 0.207 |
|  |  | Model^2^ | 0.04 (0.04) | 0.266 | -0.01 (0.03) | 0.845 | 0.04 (0.04) | 0.327 | 0.04 (0.03) | 0.231 |
| **rs12779790** | ***CDC123,CAMK1D*** | Model^1^ | 0.02 (0.04) | 0.603 | 0.01 (0.04) | 0.748 | 0.02 (0.04) | 0.731 | 0.06 (0.04) | 0.101 |
|  |  | Model^2^ | 0.02 (0.04) | 0.587 | 0.01 (0.04) | 0.742 | 0.02 (0.04) | 0.714 | 0.06 (0.04) | 0.097 |
| **rs13266634** | ***SLC30A8*** | Model^1^ | -0.02 (0.04) | 0.678 | 0.00 (0.04) | 0.921 | -0.01 (0.04) | 0.758 | -0.03 (0.03) | 0.381 |
|  |  | Model^2^ | -0.01 (0.04) | 0.708 | 0.00 (0.04) | 0.954 | -0.01 (0.04) | 0.791 | -0.03 (0.03) | 0.363 |
| **rs17044137** | ***FLJ39370*** | Model^1^ | -0.03 (0.05) | 0.537 | -0.05 (0.05) | 0.318 | -0.03 (0.05) | 0.510 | 0.00 (0.04) | 0.968 |
|  |  | Model^2^ | -0.03 (0.05) | 0.593 | -0.05 (0.05) | 0.335 | -0.03 (0.05) | 0.558 | 0.01 (0.04) | 0.894^[[5]](#footnote-5)^ |

**Table F: Examination of pleiotropic effects of lipid SNPs on glycemic traits**

|  |  | HOMA-IR | | HOMA-B | | Fasting Insulin | | Fasting Glucose | |
| --- | --- | --- | --- | --- | --- | --- | --- | --- | --- |
|  |  | **β (SE)** | **p-value** | **β (SE)** | **p-value** | **β (SE)** | **p-value** | **β (SE)** | **p-value** |
| Association between Lipid SNPs and glycemic traits | | | | | | | | | |
| rs562338^†^ | **Crude** | 0.02 (0.05) | 0.697 | 0.04 (0.04) | 0.422 | 0.03 (0.05) | 0.597 | -0.04 (0.04) | 0.302 |
| rs662799^$^ | **Crude** | 0.05 (0.04) | 0.261 | 0.09 (0.04) | 0.027^*^ | 0.06 (0.04) | 0.187 | -0.04 (0.04) | 0.257 |
| rs780094^‡^ | **Crude** | 0.07 (0.04) | 0.086 | 0.08 (0.04) | 0.031^*^ | 0.07 (0.04) | 0.076 | 0.00 (0.03) | 0.986 |
| rs4420638^‡^ | **Crude** | -0.03 (0.05) | 0.566 | -0.05 (0.05) | 0.243 | -0.04 (0.05) | 0.481 | 0.04 (0.04) | 0.334 |
| rs6511720^†^ | **Crude** | -0.07 (0.07) | 0.293 | -0.06 (0.06) | 0.371 | -0.08 (0.07) | 0.246 | 0.04 (0.06) | 0.461 |
| rs6993414^‖^ | **Crude** | -0.13 (0.05) | 0.006^*^ | -0.11 (0.05) | 0.017^*^ | -0.13 (0.05) | 0.007^*^ | -0.04 (0.04) | 0.361 |
| rs10503669^‡^ | **Crude** | -0.14 (0.05) | 0.007^*^ | -0.09 (0.05) | 0.061 | -0.14 (0.05) | 0.010^*^ | -0.07 (0.05) | 0.141 |
| Association between lipid SNPs and glycemic traits adjusted for lipid levels ^‡‡^ | | | | | | | | | |
| rs562338^§^ | **Model^1^** | 0.03 (0.05) | 0.559 | 0.04 (0.04) | 0.374 | 0.03 (0.05) | 0.482 | -0.03 (0.04) | 0.405 |
| rs662799^§^ | **Model^1^** | 0.04 (0.04) | 0.342 | 0.08 (0.04) | 0.033^v^ | 0.05 (0.04) | 0.244 | -0.05 (0.04) | 0.182 |
| rs6511720^§^ | **Model^1^** | -0.06 (0.07) | 0.373 | -0.05 (0.06) | 0.407 | -0.07 (0.07) | 0.307 | 0.05 (0.06) | 0.353 |
| rs662799^‡^ | **Model^1^** | 0.02 (0.04) | 0.649 | 0.07 (0.04) | 0.074 | 0.03 (0.04) | 0.491 | -0.06 (0.04) | 0.104 |
| rs780094^‡^ | **Model^1^** | 0.05 (0.04) | 0.174 | 0.07 (0.04) | 0.054 | 0.06 (0.04) | 0.151 | -0.01 (0.03) | 0.797 |
| rs4420638^‡^ | **Model^1^** | -0.05 (0.05) | 0.345 | -0.06 (0.05) | 0.161 | -0.05 (0.05) | 0.291 | 0.03 (0.04) | 0.463 |
| rs6993414^‡^ | **Model^1^** | -0.11 (0.05) | 0.017^*^ | -0.10 (0.05) | 0.032^††^ | -0.11 (0.05) | 0.019^*^ | -0.03 (0.04) | 0.524 |
| rs10503669^‡^ | **Model^1^** | -0.12 (0.05) | 0.021^*^ | -0.08 (0.05) | 0.108 | -0.11 (0.05) | 0.027^*^ | -0.05 (0.04) | 0.237 |
| rs6993414^¶^ | **Model^1^** | -0.14 (0.05) | 0.006^*^ | -0.11 (0.05) | 0.020^*^ | -0.13 (0.05) | 0.007^*^ | -0.04 (0.04) | 0.301 |
| rs562338^**^ | **Model^1^** | 0.02 (0.05) | 0.638 | 0.04 (0.05) | 0.422 | 0.03 (0.05) | 0.557 | -0.03 (0.04) | 0.410 |
| rs6511720^**^ | **Model^1^** | -0.06 (0.07) | 0.321 | -0.05 (0.06) | 0.415 | -0.07 (0.07) | 0.270 | 0.04 (0.06) | 0.443^[[6]](#footnote-6)^ |

**Table G: Examination of pleiotropic effects of glycemic SNPs on lipid traits**

|  | | |  | | | | Total Cholesterol | | | | | | Triglycerides | | | | | HDL-C | | | | LDL-C | | | | |
| --- | --- | --- | --- | --- | --- | --- | --- | --- | --- | --- | --- | --- | --- | --- | --- | --- | --- | --- | --- | --- | --- | --- | --- | --- | --- | --- |
|  | | |  | | | **β (SE)** | | **p-value** | | | | **β (SE)** | | **p-value** | | | | **β (SE)** | | **p-value** | | **β (SE)** | | **p-value** | | |
| Association between Glycemic SNPs and lipid traits | | | | | | | | | | | | | | | | | | | | | | | | | | |
| rs2641348^$^ | **Crude** | | | | 0.04 (0.04) | | | | | 0.342 | 0.00 (0.04) | | | | | 0.987 | | | -0.05 (0.04) | | 0.202 | | 0.06 (0.04) | | | 0.118 |
| rs10811661^†^ | **Crude** | | | | 0.00 (0.04) | | | | | 0.939 | -0.10 (0.05) | | | | | 0.029^*^ | | | 0.01 (0.04) | | 0.819 | | 0.04 (0.04) | | | 0.421 |
| rs10923931^†^ | **Crude** | | | | 0.04 (0.04) | | | | | 0.255 | 0.01 (0.04) | | | | | 0.798 | | | -0.07 (0.04) | | 0.072 | | -0.01 (0.04) | | | 0.767 |
| rs7756992^‡^ | **Crude** | | | | -0.04 (0.04) | | | | | 0.336 | 0.06 (0.04) | | | | | 0.128 | | | 0.00 (0.04) | | 0.942 | | -0.05 (0.04) | | | 0.151 |
| rs7903146^‡^ | **Crude** | | | | 0.00 (0.04) | | | | | 0.923 | 0.01 (0.04) | | | | | 0.863 | | | 0.06 (0.03) | | 0.073 | | -0.02 (0.04) | | | 0.511 |
| Association between Glycemic SNPs and lipid traits adjusted for glycemic levels ^††^ | | | | | | | | | | | | | | | | | | | | | | | | | | |
| rs2641348^‖^ | | **Model^1^** | | 0.04 (0.04) | | | | | | 0.248 | 0.01 (0.04) | | | | 0.702 | | -0.05 (0.04) | | | | 0.215 | | 0.06 (0.04) | | 0.095 | |
| rs10811661^‖^ | | **Model^1^** | | 0.00 (0.04) | | | | | | 0.929 | -0.08 (0.04) | | | | 0.057 | | 0.01 (0.04) | | | | 0.795 | | 0.04 (0.04) | | 0.367 | |
| rs10923931^‖^ | | **Model^1^** | | 0.05 (0.04) | | | | | 0.194 | | 0.02 (0.04) | | | | 0.570 | | -0.04 (0.04) | | | | 0.317 | | 0.07 (0.04) | | 0.060 | |
| rs2641348^§^ | | **Model^1^** | | 0.04 (0.04) | | | | | 0.348 | | 0.01 (0.04) | | | | 0.845 | | -0.05 (0.04) | | | | 0.16 | | 0.06 (0.04) | | 0.128 | |
| rs2641348^¶^ | | **Model^1^** | | 0.04 (0.04) | | | | | 0.255 | | 0.01 (0.04) | | | | 0.709 | | -0.05 (0.04) | | | | 0.207 | | 0.06 (0. 04) | | 0.097 | |
| rs10811661^¶^ | | **Model^1^** | | 0.00 (0.04) | | | | | 0.944 | | -0.09 (0.05) | | | | 0.055 | | 0.01 (0.04) | | | | 0.809 | | 0.04 (0.04) | | 0.374 | |
| rs10923931^¶^ | | **Model^1^** | | 0.05 (0.12) | | | | | 0.200 | | 0.02 (0.10) | | | | 0.581 | | -0.04 (0.04) | | | | 0.309 | | 0.07 (0.15) | | 0.062 | |
| rs7756992** | | **Model^1^** | | -0.04 (0.04) | | | | | 0.248 | | 0.05 (0.04) | | | | 0.186 | | -0.01 (0.04) | | | | 0.830 | | -0.06 (0.04) | | 0.123 | |
| rs7903146** | | **Model^1^** | | 0.01 (0.04) | | | | | 0.847 | | 0.00 (0.04) | | | | 0.958 | | 0.06 (0.03) | | | | 0.058 | | -0.02 (0.04) | | 0.596^[[7]](#footnote-7)^ | |

**Table H: Association between validated SNPs and confounders**

|  | **Age** | **Gender** | **Location** | **Site** | **BMI** | **Fat^#^** | **Energy^#^** | **Carbohydrate^#^** | **PA** | **Alcohol** | **Smoking** |
| --- | --- | --- | --- | --- | --- | --- | --- | --- | --- | --- | --- |
| **Lipid-Related SNPs (p values)** | | | | | | | | | | | |
| **rs662799** | 0.715 | 0.914 | 0.646 | 0.112 | 0.852 | 0.789 | 0.985 | 0.969 | 0.353 | 0.480 | 0.639 |
| **rs562338** | 0.728 | 0.247 | 0.357 | 0.714 | 0.486 | 0.026^*^ | 0.317 | 0.138 | 0.160 | 0.348 | 0.528 |
| **rs6511720** | 0.648 | 0.452 | 0.302 | 0.567 | 0.776 | 0.746 | 0.693 | 0.592 | 0.010^*^ | 0.131 | 0.894 |
| **rs780094** | 0.591 | 0.584 | 0.476 | 0.187 | 0.972 | 0.378 | 0.791 | 0.585 | 0.241 | 0.161 | 0.470 |
| **rs4420638** | 0.258 | 0.848 | 0.549 | 0.679 | 0.730 | 0.357 | 0.829 | 0.750 | 0.977 | 0.442 | 0.419 |
| **rs6993414** | 0.145 | 0.129 | 0.033^*^ | 0.123 | 0.162 | 0.235 | 0.394 | 0.328 | 0.258 | 0.782 | 0.368 |
| **rs10503669** | 0.665 | 0.639 | 0.030^*^ | 0.106 | 0.084 | 0.181 | 0.548 | 0.356 | 0.243 | 0.436 | 0.145 |
| **Glycemic-Related SNPs (p values)** | | | | | | | | | | | |
| **rs2641348** | 0.370 | 0.514 | 0.422 | 0.388 | 0.508 | 0.968 | 0.718 | 0.798 | 0.328 | 0.603 | 0.748 |
| **rs10811661** | 0.917 | 0.683 | 0.187 | 0.847 | 0.959 | 0.418 | 0.395 | 0.765 | 0.290 | 0.241 | 0.782 |
| **rs10923931** | 0.093 | 0.240 | 0.442 | 0.023^*^ | 0.954 | 0.859 | 0.381 | 0.434 | 0.289 | 0.706 | 0.986 |
| **rs7756992** | 0.674 | 0.700 | 0.443 | 0.174 | 0.346 | 0.407 | 0.412 | 0.367 | 0.134 | 0.327 | 0.026^*^ |
| **rs7903146** | 0.914 | 0.182 | 0.180 | 0.357 | 0.320 | 0.274 | 0.859 | 0.780 | 0.970 | 0.138 | 0.942^[[8]](#footnote-8)^ |

|  |  | **Assumption 1^$^** | | | **Assumption 2 (Outcome)^#^** | | | | | | | **Assumption 3 (Confounders)^@^** | | | | | | | | | | | | |
| --- | --- | --- | --- | --- | --- | --- | --- | --- | --- | --- | --- | --- | --- | --- | --- | --- | --- | --- | --- | --- | --- | --- | --- | --- |
|  |  | **ASSUMPTION CHECK FOR LIPID-RELATED INSTRUMENTS** | | | | | | | | | | | | | | | | | | | | | | |
|  | **Instrument (N)** | **Exposure** | | **H-IR** | | | **H-β** | | **FI** | | **FG** | | **1** | **2** | **3** | **4** | **5** | **6** | **7** | **8** | **9** | | **10** | **11** |
| **TC** | rs662799 + rs562338 + rs6511720  **(4552)** | 0.94 (0.26),  <0.001^*^44.82 | | 0.405, 0.584 | | | - | | 0.360, 0.505 | | 0.636, 0.418 | | 0.657 | 0.701 | 0.254 | 0.590 | 0.618 | 0.344 | 0.740 | 0.904 | 0.008 | | 0.325 | 0.505 |
| **TC** | rs562338 + rs6511720  **(4690)** | 0.98 (0.31),  0.001^*^, 43.64 | | 0.633, 0.812 | | | 0.979, 0.948 | | 0.656, 0.812 | | 0.774, 0.994 | | 0.605 | 0.69 | 0.197 | 0.410 | 0.744 | 0.198 | 0.441 | 0.581 | 0.007 | | 0.136 | 0.678 |
| **TG** | rs662799 + rs780094 + rs4420638  **(4360)** | 0.88 (0.23),  <0.001^*^, 51.69 | | 0.060, 0.213 | | | 0.018*, 0.051 | | 0.040^*^,0.167 | | 0.774, 0.459 | | 0.497 | 0.696 | 0.206 | 0.429 | 0.481 | 0.606 | 0.751 | 0.907 | 0.371 | | 0.717 | 0.559 |
| **LDL-C** | rs562338 + rs6511720  **(4690)** | 1.01 (0.31) 0.001^*^, 36.71 | | 0.650, 0.730 | | | 0.999, 0.956 | | 0.676, 0.743 | | 0.748, 0.904 | | 0.605 | 0.665 | 0.199 | 0.686 | 0.717 | 0.181 | 0.419 | 0.563 | 0.008 | | 0.139 | 0.674 |
|  |  | **ASSUMPTION CHECK FOR GLYCEMIC-RELATED INSTRUMENTS** | | | | | | | | | | | | | | | | | | | | | | |
|  | **Instrument**  **(N)** | **Exposure** | **TC** | | | **TG** | | **HDL-C** | | **LDL-C** | | | **1** | **2** | **3** | **4** | **5** | **6** | **7** | **8** | **9** | **10** | | **11** |
| **H-IR** | rs2641348 + rs10811661  **(4632)** | 1.04 (0.34), 0.001^*^, 67.00 | 0.276, 0.177 | | | 0.277, 0.548 | | 0.531, 0.562 | | 0.039^*^, 0.027^*^ | | | 0.578 | 0.825 | 0.059 | 0.652 | 0.920 | 0.684 | 0.988 | 0.784 | 0.098 | 0.769 | | 0.644 |
| **H-β** | rs2641348  **(4900)** | -0.07 (0.04), 0.049, 55.83 | 0.342, 0.348 | | | 0.987, 0.845 | | 0.202, 0.160 | | 0.118, 0.128 | | | 0.370 | 0.514 | 0.422 | 0.388 | 0.508 | 0.968 | 0.718 | 0.798 | 0.328 | 0.603 | | 0.748 |
| **FI** | rs2641348 + rs10811661  **(4632)** | 1.04 (0.31), 0.001^*^, 63.88 | 0.275, 0.183 | | | 0.289, 0.558 | | 0.516, 0.528 | | 0.039^*^, 0.029^*^ | | | 0.567 | 0.812 | 0.061 | 0.368 | 0.926 | 0.693 | 0.978 | 0.801 | 0.098 | 0.784 | | 0.645 |
| **FG** | rs7756992 + rs7903146  **(4748)** | 0.98 (0.31), 0.001^*^, 70.92 | 0.566, 0.410 | | | 0.123, 0.196 | | 0.280, 0.367 | | 0.140, 0.107 | | | 0.758 | 0.258 | 0.865 | 0.117 | 0.311 | 0.268 | 0.45 | 0.629 | 0.193 | 0.086 | | 0.086^[[9]](#footnote-9)^ |

**Table I: Summary of MR assumptions of the instruments (wGRS) generated from validated SNPs**

**Table J: Durbin-Hausman Test for association between lipids and glycemic traits**

|  | Glycemic (Independent) Lipid(Dependent) | | | | | Lipid (Independent) Glycemic(Dependent) | | | |
| --- | --- | --- | --- | --- | --- | --- | --- | --- | --- |
|  | **HOMA-IR** | | **HOMA-β** | **Fasting Insulin** | **Fasting Glucose** | **HOMA-IR** | **HOMA-β** | **Fasting Insulin** | **Fasting Glucose** |
|  | **p-value** | **p-value** | | **p-value** | **p-value** | **p-value** | **p-value** | **p-value** | **p-value** |
| Total Cholesterol | 0.978^*^ | 0.986^*^ | | 0.975^*^ | 0.991 | 0.999^†^ | - | 0.001^†^ | 0.997^†^ |
|  |  |  | |  |  | <0.001^‡^ | <0.001^‡^ | <0.001^‡^ | 0.842^‡^ |
| Triglycerides | 0.997 | 0.97 | | 0.998 | 0.937 | 0.021 | <0.001 | 0.023 | 0.971 |
| HDL-C | 0.003 | <0.001 | | <0.001 | 0.986 | - | - | - | - |
| LDL-C | - | - | | - | 0.431 | <0.001 | <0.001 | <0.001 | 0.621^[[10]](#footnote-10)^ |

**Table K: MR Assumption checks of different instruments of triglycerides that showed causal association with glycemic traits**

|  | Assumption 1 (Exposure) ^$^ | Assumption 2 (Outcome)^#^ | | | | Assumption 3 (Confounders)^@^ | | | | | | | | | | |
| --- | --- | --- | --- | --- | --- | --- | --- | --- | --- | --- | --- | --- | --- | --- | --- | --- |
| Instrument,  (N) | **β (SE), p,**  **F-Statistics** | **HOMA-IR** | **HOMA-β** | **FI** | **FG** | **1** | **2** | **3** | **4** | **5** | **6** | **7** | **8** | **9** | **10** | **11** |
| rs662799 + rs780094  4684 | 0.89 (0.26),0.001^*^, 50.19 | 0.017^*^, 0.063 | 0.001^*^, 0.003 | 0.009^*^, 0.037^*^ | 0.343, 0.177 | 0.020^*^ | 0.439 | 0.212 | 0.712 | 0.282 | 0.743 | 0.613 | 0.585 | 0.352 | 0.365 | 0.451 |
| rs662799 + rs4420638  4682 | 0.96 (0.26), <0.001^*^, 50.14 | 0.171, 0.457 | 0.145, 0.292 | 0.164, 0.423 | 0.813, 0.838 | 0.442 | 0.919 | 0.279 | 0.424 | 0.832 | 0.517 | 0.722 | 0.942 | 0.370 | 0.771 | 0.882 |
| rs780094 + rs4420638  4726 | 0.98 (0.36)  0.007^*^, 50.96 | 0.432, 0.780 | 0.454, 0.674 | 0.453, 0.789 | 0.582, 0.826 | 0.205 | 0.642 | 0.441 | 0.224 | 0.941 | 0.928 | 0.904 | 0.753 | 0.373 | 0.641 | 0.807 |
| rs662799  4820 | 0.17 (0.04), <0.001^*^, 22.97 | 0.261, 0.649 | 0.027^*^, 0.074 | 0.187, 0.491 | 0.257, 0.104 | 0.715 | 0.914 | 0.646 | 0.112 | 0.852 | 0.789 | 0.985 | 0.969 | 0.353 | 0.480 | 0.639 |
| rs780094  4864 | 0.08 (0.04),  0.048, 16.95 | 0.086, 0.174 | 0.031^*^**,** 0.054 | 0.076, 0.151 | 0.986, 0.797 | 0.591 | 0.584 | 0.476 | 0.187 | 0.972 | 0.378 | 0.791 | 0.585 | 0.241 | 0.161 | 0.470 |
| rs4420638  4820 | 0.10 (0.05), 0.048^*^, 16.85 | 0.566, 0.345 | 0.243, 0.161 | 0.481, 0.291 | 0.334, 0.463 | 0.258 | 0.848 | 0.549 | 0.679 | 0.730 | 0.357 | 0.829 | 0.750 | 0.977 | 0.442 | 0.419^[[11]](#footnote-11)^ |

1. **Model^1^ - Adjusted for Age, sex, site, location and BMI**

   **Model^2^ - Adjusted for Age, sex, site, location, BMI, average daily fat intake, average daily energy intake, average daily carbohydrate intake, MET Score/day, alcohol consumption and smoking**

   **β (SE) – Standardized coefficients i.e SD unit change in outcome per SD unit increase in exposure**

   ^*^**Level of significance p< 0.05** [↑](#footnote-ref-1)
2. **Model^1^ - Adjusted for Age, sex, site, location and BMI**

   **Model^2^ - Adjusted for Age, sex, site, location, BMI, average daily fat intake, average daily energy intake, average daily carbohydrate intake, MET Score/day, alcohol consumption and smoking**

   **β (SE) – Standardized coefficients i.e SD unit change in outcome per SD unit increase in exposure**

   ^*^**Level of significance p< 0.05** [↑](#footnote-ref-2)
3. **^*^Significance Threshold after Multiple Testing Correction (Sidak Correction) p= 0.0011**

   **Any SNP with lower significance deviates from HWE**

   **^#^ HWE tested on unrelated participants** [↑](#footnote-ref-3)
4. **Model^1^ - Adjusted for Age, sex, site, location and BMI**

   **Model^2^ - Adjusted for Age, sex, site, location, BMI, average daily fat intake, average daily energy intake, average daily carbohydrate intake, MET Score/day, alcohol consumption and smoking**

   **β (SE) – Standardized coefficients i.e. SD unit change in lipid per risk allele**

   **^*^ Level of significance p< 0.05** [↑](#footnote-ref-4)
5. **Model^1^- Adjusted for Age, sex, site, location and BMI**

   **Model^2^ - Adjusted for Age, sex, site, location, BMI, average daily fat intake, average daily energy intake, average daily carbohydrate intake, MET Score/day, alcohol consumption and smoking**

   **β (SE) – Standardized coefficients i.e. SD unit change in lipid per risk**

   **^*^Level of significance p<0.05** [↑](#footnote-ref-5)
6. **Step 1: Crude; †, ^$^, ‡ and ‖ represents SNPs associated with (TC, LDL-C), (TC, TG), TG and (TG, HDL-C) respectively**

   **Step 2: Model^1^ – ^§^Adjusted for TC, ^‡^Adjusted for TG, ^¶^Adjusted for HDL-C, ^**^Adjusted for LDL-C**

   **TC-Total Cholesterol, TG –Triglycerides**

   **β (SE) – Standardized coefficients i.e SD unit change in outcome per SD unit increase in exposure**

   **^*^Level of significance p< 0.05**

   **^‡‡^Significant association in this step will lead to elimination of the variants from any further analyses** [↑](#footnote-ref-6)
7. **Step 1: Crude, $, † and ‡ represents SNPs associated with (HOMA-IR, HOMA-β, FI), (HOMA-IR, FI) and FG respectively**

   **Step 2: Model^1^ – ‖ - Adjusted for HOMA-IR, § - Adjusted for HOMA–β, ¶ - Adjusted for FI, ** - Adjusted for FG**

   **FI–Fasting Insulin, FG–Fasting Glucose**

   **β (SE) – Standardized coefficients i.e SD unit change in outcome per SD unit increase in exposure**

   ***Level of significance p< 0.05**

   **††Significant association in this step will lead to elimination of the variants from any further analyses** [↑](#footnote-ref-7)
8. **BMI – Body Mass Index, PA – Physical Activity**

   **^#^Dietary factors include daily fat intake, daily energy intake and daily carbohydrate intake**

   **^*^Level of significance p<0.05** [↑](#footnote-ref-8)
9. H-**β = HOMA-β, H-IR = HOMA-IR, FI = Fasting Insulin, FG = Fasting Glucose, TC = Total Cholesterol, TG = Triglycerides**

   **^*^Level of significance p<0.05**

   **Assumption 1^$^ – β(SE), p-value, F statistics**

   **Assumption 2 (Outcome)^#^ – p1-value of crude model, p2-value of model adjusted for respective exposure**

   **Assumption 3 (Confounder)^@^ – p-value of model run for assessment of association between variant and respective confounder**

   **1-Age, 2- Gender, 3- Location, 4 – Site, 5- BMI, 6- Daily Fat Intake, 7 – Daily Energy Intake, 8 – Daily Carbohydrate Intake, 9 – Physical Activity, 10 – Alcohol Consumption, 11 – Smoking** [↑](#footnote-ref-9)
10. ***Comparison between effect of phenotypic glycemic levels and glycemic trait explained by instruments on cholesterol (outcome)**

    **†Comparison between effects of phenotypic cholesterol and cholesterol explained by single SNP**

    **‡Comparison between effects of phenotypic cholesterol and cholesterol explained by combination of SNP**

    **- indicates that MR analysis was not performed due to violation of MR assumptions**

    **p-value from Durbin-Hausman test which compares the difference between estimates derived from the OLS and 2 SLS regressions** [↑](#footnote-ref-10)
11. **^*^Level if significance p<0.05**

    **Assumption 1^$^ – β(SE), p-value, F statistics**

    **Assumption 2 (Outcome)^#^ – p1-value of crude model, p2-value of model adjusted for respective exposure**

    **Assumption 3 (Confounder)^@^ – p-value of model run for assessment of association between variant and respective confounder**

    **1-Age, 2- Gender, 3- Location, 4 – Site, 5- BMI, 6- Daily Fat Intake, 7 – Daily Energy Intake, 8 – Daily Carbohydrate Intake, 9 – Physical Activity, 10 – Alcohol Consumption, 11 – Smoking** [↑](#footnote-ref-11)
